# Myocardial Infarction Induced by Refrigerant (Hydrofluorocarbon (HFC)-134a) Exposure: A Case Report

**DOI:** 10.7759/cureus.81706

**Published:** 2025-04-04

**Authors:** Christian Roberti, Margaret Mueller

**Affiliations:** 1 Medicine, Lake Erie College of Osteopathic Medicine, Bradenton, USA; 2 Emergency Medicine, Sturdy Memorial Hospital, Attleboro, USA

**Keywords:** freon, heart attack, mi, myocardial infarction, refrigerant

## Abstract

Refrigerants such as hydrofluorocarbon (HFC)-134a (1,1,1,2-tetrafluoroethane) are widely used in refrigeration and air-conditioning systems. While their toxic effects are generally considered low, cardiotoxicity associated with arrhythmias has been reported. However, to the best of our knowledge, myocardial infarction (MI) immediately following refrigerant exposure has not been documented in the literature until now.

We report the case of a 75-year-old man with coronary artery disease, an assistant professor at a community college specializing in refrigeration technology and heating, ventilation, and air conditioning (HVAC) standards. He was exposed to HFC-134a while repairing a leak in an antique refrigeration unit when he experienced a sudden onset of dyspnea and mild chest pain. He presented to the emergency department, where workup revealed worsening T-wave abnormalities compared to an electrocardiogram (EKG) taken two months prior, along with elevated troponin levels, confirming an acute MI. The patient was placed on supplemental oxygen and stabilized with antihypertensives and nitroglycerin.

This case highlights a potential association between acute refrigerant exposure and MI, a novel finding not previously reported. Possible mechanisms include cardiac sensitization to epinephrine or hypoxia-induced ischemia, although acute stress-related MI cannot be ruled out, especially with the patient's underlying conditions. Further research is needed to explore the cardiotoxic potential of refrigerants, particularly in individuals with preexisting coronary artery disease.

## Introduction

Hydrofluorocarbon (HFC)-134a (1,1,1,2-tetrafluoroethane) is a synthetic refrigerant used widely in air conditioning and refrigeration systems. It is a synthetic carbon compound containing hydrogen and fluorine substituents that has minimal reported toxicity. The only documented cardiotoxic effects related to substantial concentrations of this gas are cardiac arrhythmias [1,2]. In this report, we present an unusual case of acute myocardial infarction (MI) secondary to refrigerant exposure in a patient with prior coronary artery stenting.

## Case presentation

The patient is a 75-year-old man with a history of smoking, hypertension, chronic obstructive pulmonary disease (COPD), hyperlipidemia, obesity, and coronary artery disease status post stenting. He presented to the emergency department via emergency medical services (EMS) with a chief complaint of profound dyspnea and mild chest pain associated with refrigerant exposure. The patient stated that he had accidentally inhaled the refrigerant, commercially known as R134a, while repairing a refrigerant line in an antique refrigerator. During exposure, he was in a small, enclosed area, allowing the refrigerant gases to reach peak atmospheric concentrations.

**Figure 1:**
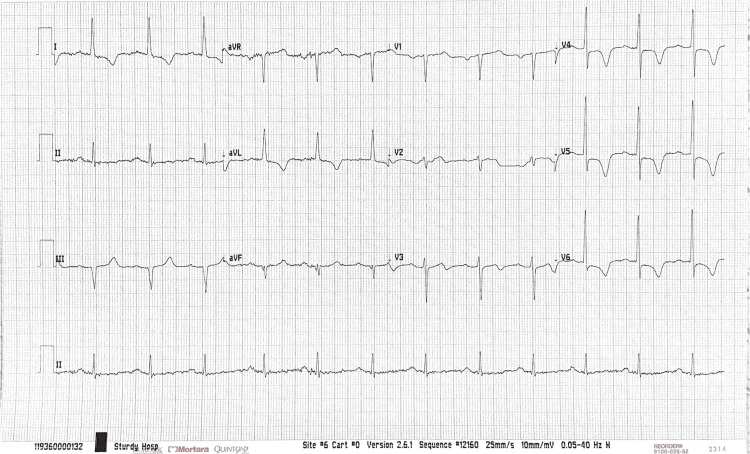
Electrocardiogram (EKG) Findings

Upon arrival at the emergency department, the patient’s symptoms of chest pressure, shortness of breath, and angina had resolved, and his oxygen saturation increased from 94% upon EMS arrival to 96% in the emergency department after being placed on 2L nasal cannula. Differential diagnoses included, but were not limited to, reactive airways disease and angina. Emergent chest X-rays were unremarkable for any intrathoracic abnormalities, while the current electrocardiogram (EKG) (Figure 1) revealed inferior Q waves consistent with a previous MI, left ventricular hypertrophy (LVH), and first-degree atrioventricular (AV) block, comparable to his EKG two months prior. However, there was worsening T-wave inversion in leads I, aVL, and V2-V6. The patient’s blood pressure was 205/110, which decreased to 174/107 following the administration of 10 mg of IV labetalol. Initial troponin levels were 53 ng/L, with a repeat troponin of 76 ng/L (Δ23), consistent with a non-ST-segment elevation MI. Emergent IV nitroglycerin drip was started to ensure adequate myocardial blood flow, and the patient was admitted. The patient was prescribed rosuvastatin 40 mg daily and 0.4 mg nitroglycerin sublingual tablets to take as needed if his angina persisted.

## Discussion

HFC-134a, a halogenated and fluorinated hydrocarbon, is a volatile gas used in refrigeration and air-conditioning and is commercially known as Freon. Its toxicity in both human and animal subjects has been documented as very low, though experimental studies show increased cardiac sensitization to epinephrine when HFC-134a is administered in high concentrations to animal models [1]. This cardiac sensitization is a proposed mechanism for the development of arrhythmias, the most common cardiac abnormality associated with refrigerant inhalation, although other hypotheses exist, such as sinoatrial (SA) node depression with subsequent ectopic escape rhythms [2]. It has been proposed that this sensitization is positively correlated with the degree of chlorine and fluorine substitutions on the hydrocarbon chain, although the exact molecular dynamics of this mechanism remain unclear. Although HFC-134a is not chlorinated, it does contain fluorine substituents. Furthermore, in the same study, cardiac sensitization was not reported in humans when HFC-134a was administered as an inhaler propellant due to lower experimental dosing. When MIs were experimentally induced in a dog heart model, cardiac sensitization remained unchanged relative to the undamaged dog heart [1].

In another study analyzing the cardiotoxicity of HFCs among refrigeration workers, the only reported cardiac symptoms were arrhythmias, consistent with the previous study on refrigerant-induced cardiac sensitization [2]. However, unlike our patient, none of the subjects in that study had signs of coronary artery disease. Conversely, a similar study that sought to correlate arrhythmias with HFC exposure found no clear connection [3]. However, the subjects in that study were exposed to chlorodifluoromethane (FC-22) and dichlorodifluoromethane (FC-12) over an extended time interval rather than in an acute setting, making the results inconclusive relative to the present case.

Although no dysrhythmia was documented in our patient's case, it is possible that he experienced a transient dysrhythmia secondary to cardiac sensitization, which may have contributed to his ischemia. This dysrhythmia could have occurred at the onset of his symptoms and resolved before EMS arrival, leading to unremarkable EKG recordings. The patient had reported improvement in his symptoms when EMS first arrived.

Another potential mechanism is hypoxia-induced ischemia. A case report published on a previously healthy 19-year old air conditioning technician described acute onset shortness of breath, dyspnea, and chest tightness following exposure to HFC during a refrigerant gas leak in a confined space. The patient was hospitalized, and high-resolution computed tomography of the chest revealed bilateral pulmonary infiltrates, leading to a diagnosis of HFC pneumonitis [4].

The symptomatic chronology of pneumonitis in this patient closely resembles this case, although no chest imaging was conducted. Pneumonitis in our patient's case could have caused acute cardiac ischemia. While his oxygen saturation was recorded at 94% upon EMS evaluation, and he noted an improvement in his breathing, it is possible that he experienced more severe hypoxia when his symptoms initially began.

Although the exact atmospheric concentration of HFC-134a at the time of incidence is unknown, exposure guidelines proposed by the National Research Council recommend a maximum of 4,000 ppm per one hour exposure. This recommendation was based on cardiac sensitization induced in male beagles when exposed to HFC at different levels over a one-hour period. The current exposure guidelines based on this experiment for different time intervals can be found in Table 1 [5].

**Table 1:** Proposed Recommendations for HFC-134a by the Subcommittee EEGL: Emergency exposure guideline level; CEGL: Continuous exposure guideline level; HFC: Hydrofluorocarbon

Exposure	Concentration (ppm)
1-hr EEGL	4,000
24-hr EEGL	1,000
90-day CEGL	900

MI induced by refrigerant inhalation is a rare occurrence, with minimal documented case reports describing a similar clinical presentation. Further research is needed to better understand the cardiotoxicity related to refrigerant exposure. The MI we present in this case could have been due to the arrhythmogenic properties of this gas or hypoxia secondary to localized lung irritation. A possible limitation of this study, however, is the inability to rule out underlying comorbidities as causative or contributing factors to these circumstances. Given the patient’s known coronary artery disease, he was at increased risk for myocardial ischemia. However, given the temporal relationship between exposure and symptoms, an association between refrigerant exposure and MI is highly suggested.

## Conclusions

Regardless of whether this MI was caused by the refrigerant-induced arrhythmia or by hypoxia, proper safety precautions should be employed when working with such gases. Our patient was exposed to a poorly ventilated area without proper protective equipment, which led to his cardiac emergency. Further research is needed to determine the association between HFC-134a exposure and MI and whether certain safety precautions can prevent such an event. However, it seems evident that improper ventilation is a key risk factor in developing acute toxicity, thus allowing gas concentration to exceed the maximum exposure levels. With this in mind, we urge anyone working with HFC-134a or similar refrigerants to follow appropriate safety precautions and current exposure guidelines to prevent life-threatening emergencies.

## References

[1] National Research Council (US) Subcommittee on Acute Exposure Guideline Levels (2002). 1,1,1,2-tetrafluoroethane (HFC-134a)1: acute exposure guideline levels. Acute Exposure Guideline Levels for Selected Airborne Chemicals: Volume 2.

[2] Sabik LM, Abbas RA, Ismail MM, El-Refaei S (2009). Cardiotoxicity of Freon among refrigeration services workers: comparative cross-sectional study. Environ Health.

[3] Antti-Poika M, Heikkilä J, Saarinen L (1990). Cardiac arrhythmias during occupational exposure to fluorinated hydrocarbons. Br J Ind Med.

[4] Abdul Malik AA, Ng BH, Nik Abeed NN, Abdul Hamid MF, Ban AY (2022). Hydrofluorocarbons pneumonitis as a complication of inhalation injury following air-conditioning repairs. Respirol Case Rep.

[5] National Research Council (US) Subcommittee to Review Toxicity of Alternatives to Chlorofluorocarbons (1996). Toxicity of Alternatives to Chlorofluorocarbons: HFC-134a and HCFC-123. Toxicity of Alternatives to Chlorofluorocarbons: HFC-134a and HCFC-123.

